# Reflections on the Unintended Consequences of the Promotion of Institutional Pregnancy and Birth Care in Burkina Faso

**DOI:** 10.1371/journal.pone.0156503

**Published:** 2016-06-03

**Authors:** Andrea Melberg, Abdoulaye Hama Diallo, Ana Lorena Ruano, Thorkild Tylleskär, Karen Marie Moland

**Affiliations:** 1 Centre for International Health, Department of Global Public Health and Primary Care, University of Bergen, Bergen, Norway; 2 Centre MURAZ, Ministère de la Santé, Bobo-Dioulasso, Burkina Faso; 3 Department of Public Health, UFR-SDS, University of Ouagadougou, Ouagadougou, Burkina Faso; 4 Center for the Study of Equity and Governance in Health Systems, Guatamala city, Guatemala; 5 Centre for Intervention Science in Maternal and Child Health (CISMAC), University of Bergen, Bergen, Norway; Centre for Geographic Medicine Research Coast, KENYA

## Abstract

The policy of institutional delivery has been the cornerstone of actions aimed at monitoring and achieving MDG 5. Efforts to increase institutional births have been implemented worldwide within different cultural and health systems settings. This paper explores how communities in rural Burkina Faso perceive the promotion and delivery of facility pregnancy and birth care, and how this promotion influences health-seeking behaviour. A qualitative study was conducted in South-Western Burkina Faso between September 2011 and January 2012. A total of 21 in-depth interviews and 8 focus group discussions with women who had given birth recently and community members were conducted. The data were analyzed using qualitative content analysis and interpreted through Merton’s concept of unintended consequences of purposive social action. The study found that community members experienced a strong pressure to give birth in a health facility and perceived health workers to define institutional birth as the only acceptable option. Women and their families experienced verbal, economic and administrative sanctions if they did not attend services and adhered to health worker recommendations, and reported that they felt incapable of questioning health workers’ knowledge and practices. Women who for social and economic reasons had limited access to health facilities found that the sanctions came with increased cost for health services, led to social stigma and acted as additional barriers to seek skilled care at birth. The study demonstrates how the global and national policy of skilled pregnancy and birth care can occur in unintentional ways in local settings. The promotion of institutional care during pregnancy and at birth in the study area compromised health system trust and equal access to care. The pressure to use facility care and the sanctions experienced by women not complying may further marginalize women with poor access to facility care and contribute to worsened health outcomes.

## Background

Despite a steady decline over the past few decades, maternal mortality continues to be a global health concern: an estimated 303, 000 women died from pregnancy-related complications in 2015, the majority living in resource poor settings [1]. The Millennium Development Goals (MDGs) sought to address and channel support for women’s health through MDG 5, aiming to reduce the burden of maternal deaths by three-quarters by 2015 compared to the 1990 figures [2]. Because measuring maternal mortality is difficult, the proportion of women giving birth with skilled health personnel has been used as an indicator for progress towards MDG 5 [3].

The policy of institutional delivery aims to provide every woman with skilled attendance at birth, and has become the cornerstone of actions aimed at monitoring and achieving MDG 5 [4,5]. Giving birth in health facilities is generally accepted to equate with skilled attendance, and be the most effective measure to reduce maternal and early neonatal mortality [4,6]. Identifying and implementing feasible strategies to increase the proportion of women giving birth in a health facility remains a major concern for researchers and policymakers on the global, national and local levels. The strategies to improve the access to and utilization of facility delivery services include distance and costs, but also improved user satisfaction as a result of enhanced quality of care within the facilities [6,7].

Efforts to increase institutional births have been implemented worldwide, and constitute a global social action that aims to reduce maternal deaths. The focus on institutional birth care has been criticized for its unintended impacts on policies and practices at global, national and local levels [8,9]. Merton’s classic paper “The unanticipated consequences of purposive social action” provides a framework to guide our understanding and analysis of some unexpected effects of MDG 5 [10]. Unintended implications of the institutional birthing policy can be divided into consequences for actors, such as the health system, patients and their families, and also for communities as a whole, since social actions have the potential to influence social structures and cultures [11]. Among the unwanted effects on the global level is the narrowing from a broad agenda of sexual and reproductive health rights to the number of institutional births [8]. Nationally, this goal has been criticized for obscuring the quality of care provided when women give birth in health facilities. At local levels, the pressure to meet performance indicators and report increased numbers of institutional deliveries has pushed health workers to report incorrect data and led to efforts to convince women to use the services ‘correctly’ [9,12].

In the wake of MDG 5, the research agenda has focused on how to increase the number of facility births, but has to a lesser degree explored users’ experiences and perceptions of the promotion of the institutional birthing policies: How are these institutional care policies promoted to users and how do communities experience this promotion? Furthermore, what are the potential implications for women who do not comply with the norm of institutional care? This paper explores how communities in rural Burkina Faso perceive the promotion and delivery of facility pregnancy and birth care, and how this promotion is perceived to influence healthcare-seeking behaviour.

## Subjects and Methods

### Study setting

Situated in West Africa, Burkina Faso is among the world’s poorest countries and has a high burden of maternal deaths, with an estimated maternal mortality ratio of 400 per 100 000 live births in 2013 [13]. In Burkina Faso births with skilled attendants take place in health facilities with few exceptions. Hence, the promotion of facility care has been the core effort aiming to reduce maternal mortality. A primary objective in the Ministry of Health’s (MoH) strategic plan to reduce maternal mortality is to increase the proportion of women giving birth with skilled assistance from 50 to 80% between 2006 and 2015 [14]. Among the factors that limit the utilization of facility care during pregnancy and at birth in Burkina Faso are distance to the health facility, financial constraints, and women’s limited decision-making power [15–17]. In this context, a subsidiary policy for pregnancy and birth care has been implemented since 2006 to reduce financial barriers to facility care [14,18]. Poor quality of care in primary health facilities has also been proposed as an explanation of frequent home births, nevertheless users’ assessment of care remains largely favourable [19–22].

The study was conducted in two health districts in the South-Western part of Burkina Faso, Banfora and Mangodara. The annual number of expected deliveries for these health districts in 2011 was 24 500 for a population of approximately 500 000 [23]. The proportion of deliveries taking place with a skilled attendant was 67% in Banfora and 59% in Mangodara [23]. At the time of the study, the area had 39 primary health centres (*Centres de santé et de promotion sociale*, *CSPS*) and one regional referral hospital in Banfora town. In the study area, subsistence farming is prevalent and maternal literacy remains very low. A cohort study among pregnant women in the area indicated that 83% had never attended school [24]. The main spoken language is Dioula.

### Data collection

The data collection lasted from September 2011 to January 2012, as part of a study on the quality of facility birth care in four health centres in the Banfora region. Assuming that facility care would differ between urban and rural areas and also taking into consideration the monthly number of births, one urban, one semi-urban and two rural facilities were purposively selected to achieve maximum diversity. According to health district data, the health centres had an assisted delivery rate varying from 48 to 77% [25,26]. The health centres varied in size, and had from 2–12 health workers with different levels of training. Their infrastructure also varied substantially; some had electricity and running water, while others relied on torches as the only source of light; and water was provided from wells situated up to one kilometre from the health centre.

A total of 21 in-depth interviews (IDIs) and 8 focus group discussions (FGDs) with women who recently experienced childbirth, their partners and community members were conducted, Table 1. A research assistant trained in sociology and fluent in Dioula and French recruited the participants in the IDIs and FGDs. She was assisted by community health workers in semi-urban and rural communities in the areas covered by the four health centres. Participants were purposively selected for the interviews, on the basis that they or their partner had given birth within the last three months. The age of the interviewees ranged from 18 to 42 years, they had none to 13 living children, and lived from one to 20 km from their local health centre. A good majority relied on subsistence farming, and only a handful had attended school. The recruitment of informants ended at the point of data saturation.

**Table 1:** Overview of IDIs and FDGs.

**In-depth Interviews: (A total of 21)**	13 women with a recent health centre birth
	5 women with a recent home birth
	3 partners
**Focus group discussions: (A total of 8)**	4 groups with female participants
	3 groups with male participants
	1 mixed-sex group

Both IDIs and FGDs were conducted in Dioula; AM conducted the IDIs with the research assistant as an interpreter, while the research assistant facilitated the FGDs in Dioula with AM as an observer. The IDIs took place in the interviewees’ home, while the FGDs took place outdoor in a public place in the community where the participants lived. Both IDIs and FGDs lasted between 45 and 90 minutes.

The interview guides included open-ended questions about practices during pregnancy and childbirth, the place of birthing and the personal, as well as community perceptions on the care provided in the health centres, S1–S3 Interview Guides. The co-authors contributed to the development of the interview guides, which were translated from French to Dioula by a certified Dioula translator. Both IDIs and FGDs were recorded and transcribed verbatim in Dioula before translation into French.

### Data analysis

During fieldwork, AM carefully read the transcripts and discussed the meaning of the verbatim transcripts and the culturally embedded expressions with the research assistant. After data collection, the transcripts were examined by drawing upon qualitative content analysis [27]. After familiarization with the dataset, initial codes were identified in the interviews. These codes were grouped into categories and subsequently into themes. For example, the quote ‘If you don’t do the weighing [attend antenatal care (ANC)] she [health worker] will say “Why haven’t you come to be weighed [attended ANC]. It’s when your child is sick you’re coming” She growls like that. She will care for you, but she will disrespect you while caring for you.’ will be grouped into the category *imposing a sanction by use of verbal reprimands* and consequently into the theme *sanctions for not using the pregnancy and childbirth services as prescribed*.

### Ethical approval

Ethical clearance was provided by the national health research ethics committee of the Ministry of Health, Burkina Faso (Ref 2011-9-57, Comité d’éthique pour la Recherche en Santé, Ministère de la Santé, Ouagadougou, Burkina Faso). The Banfora regional health directorate Chief and the Heads of Banfora and Magodara health districts provided administrative authorisations. As a great majority of the study participants were illiterate, the research assistant would read a written consent form in Dioula before signed or thumb-printed informed consent was obtained from all interviewees. When names have been used, these were changed to preserve anonymity.

## Findings

During analysis, three main themes emerged: 1) The health centre as the only place to give birth, 2) Sanctions towards women not using the pregnancy and childbirth services as prescribed, and 3) Communities as incapable of questioning health worker practices.

### ‘There is only one place to give birth’

When enquiring about possible places to give birth, participants started out by only mentioning the nearby health centre, and underlined the fact that it was the only place a woman could give birth in the area. This also applied for women having had a recent home birth, and for women that would prefer to give birth at home for their future pregnancies. Study participants stated that the authorities, health workers and community members discouraged home births. Many stated that all pregnant women had an obligation to give birth at the health centre.

‘We shouldn’t give birth at home. A pregnant woman has to go to the health centre. For all my pregnancies I have been weighed four times [attended ANC] and given birth at the health centre.’

Woman, IDI 8

According to participants, the nature of births had changed, with more complications, and a greater need for assistance nowadays. Home births were described as a result of not being able to deliver at the health centre for the following reasons: the labour was short, it was far to nearest facility, there was no access to transportation, and no money to pay for the hospitalization fees and medical prescriptions. Over time, facility births had become more available and common: giving birth at home was associated with the practices of older generations, regarded now as out of date. Many also mentioned how the reduced costs of facility births over the last few years facilitated access to care. Even though ANC was supposed to be free of charge and the price of a health centre delivery was officially 900 FCFA (1.4€) according to a newly implemented subsidiary policy, participants typically would report that they were charged 200 FCFA (0.3€) per ANC and 2000 FCFA (3€) for a facility delivery.

‘Before there were some people doing deliveries at home, but now the authorities have told us that if your wife is pregnant, you should do everything to bring her to the health centre to give birth.’

Man, FGD 2

Home births were associated with complications and participants linked home births to higher risks for both mothers and new-borns. This was explained by delayed detection and treatment of potential complications at home, but also by home births in themselves leading to complications. The participants stated that if the birth was not normal or complications occurred, help could only be sought at the health centre, where you could be referred to the regional referral hospital if necessary.

### ‘They impose sanctions on you’

Health workers were reported to enforce the use of pregnancy and childbirth services by the use of different sanctions. These varied from health centre to health centre, and AM perceived them as local incentives rather than strategies implemented at the health district level. According to participants, health workers emphasized the importance of women going for four antenatal care visits and coming at an early stage of labour to reduce the risk of birth complications. Health workers enforced the prescribed use of the pregnancy and childbirth services at the health centre through verbal reprimands as well as economic and administrative sanctions. Although informants generally seemed dissatisfied with these sanctions, a number of women recently having facility births thought they were well justified.

The fear of verbal reprimands motivated the use of facility care since not complying with the recommended antenatal care visits during pregnancy would lead to verbal abuse. In addition to potential health benefits, it was seen as important to follow health personnel’s recommendations to receive good treatment and avoid verbal reprimands. Women giving birth at home expressed how the woman, her husband and other family members accompanying her would be reprimanded when arriving at the health centre after a home birth or without having attended antenatal consultations. This was perceived as a burden in addition to not receiving facility care:

‘There are no advantages of giving birth at home. It is caused by the distance. If you go to the health centre [after a home birth] they bother you, they insult you. It is like your baby is a problem for them.’

Woman, FGD 3

Our informants reported that they experienced being blamed for poor health outcomes since the health workers tended to explain birth complication in terms of patient or community behaviour:

‘They tell us that our wives have to be weighed [attend ANC]. That we refuse our wives to leave home to go and get weighed [attend ANC]. That’s why, they say, when our wives give birth, the births get complicated. But the health centre is far. That is why [our wives don’t attend ANC].’

Man, FGD 4

Participants described economic sanctions against patients that did not comply with recommendations. Several reported that women who came to the health centre after home births were asked to pay more than women who gave birth there. This was either done by charging the services directly at a higher price, by prescribing medications or costly additional exams.

Another example concerns emergency transportation. Some participants, especially males, perceived that health workers would be more inclined to refer women to the regional hospital if they had not adhered to the recommended antenatal care in pregnancy. This led to substantially higher costs, and was seen as a punishment in some cases. Although participants perceived costly additional tests/examinations, prescriptions and referral to the regional hospital as sanctions, our data does not allow us to argue that the interventions represented an expression of health workers’ sanctioning unwanted behaviour rather than ordering medically justified interventions. However it does point to a problem of distrust in health workers. In addition, patients not complying with the antenatal care recommendations did not benefit from the free evacuation policy in case of complications:

‘If the woman has been weighed [attended ANC] and gives birth to a premature and she has to be transferred to Banfora [regional hospital] it will not be a problem. The health centre will pay [the ambulance] … If she hasn’t been weighed [attended ANC], and she gives birth to a premature at home and you bring them to the health centre. To be transferred to Banfora [regional hospital] they will charge you 15 000 francs [22.5€].’

Male, FGD 4

Administrative sanctions were also put forward as a reason to give birth at the health centre, especially for the obtainment of birth certificates. These are essential for future health care, schooling and identification documents. Several participants stated that not giving birth at the health centre or not having the antenatal care booklet would make it impossible to get the child’s birth certificate.

Economic, administrative and verbal sanctions were in some cases perceived to prevent women from seeking services, even when they were in need of facility care. The use of services as prescribed was regarded as an insurance in order to receive respectful and reasonably priced treatment if complications occurred during childbirth or the mother or child were to fall sick afterwards.

‘If a husband did not have the money to pay for the weighing [ANC], and the woman goes to the health centre to give birth, the health workers will growl at her because she did not attend the antenatal consultations. They will neglect her. If somebody sees that, they will not go to the health centre to give birth, but rather give birth at home.’

Male, FGD 2

The non-use of services due to fear of sanctions was exemplified by the particular case of Aïcha, a young single woman. While the reasons she did not attend the antenatal consultations were unclear, she wished to give birth at the health centre. Her father’s fear of being disrespected by health workers was reported as the obstacle for a health centre delivery:

‘But if you don’t drink water [attend ANC] you cannot give birth at the health centre. That’s why’…

‘You cannot go because of the lack of respect. I was told to go, but my father said no since I hadn’t gone to drink water [attend ANC]. He did not want us to go, as it would cause him disrespect at the health centre.’

Woman, IDI 16

### ‘If I say that you’re not doing a good job… will you care for me?’

Communities valued the services provided in health centres, but felt unable to evaluate them. When asked directly how they appreciated the services provided, participants were positive, although some were reluctant to evaluate the care that they received. Participants did not consider themselves competent enough, and that it was not the patient’s role to evaluate the services, considering the superior knowledge and training of health workers. During ANCs, birth complications were said to be prevented by early detection and treatment of malposition of the baby as well as sickness of the mother and foetus. During birth, the advantages were related to detecting complications, the possibility of receiving injections to make the labour shorter and less painful and clean cutting of the cord. Access to neonatal vaccines was also one of the benefits mentioned in receiving care at the health centre.

‘If you go there [the health centre], they give you a needle to give birth quickly. But at home you don’t have that. That’s why we want to go [to the health centre].’

Woman, IDI 9

For communities situated far away from a health facility, having a health centre closer to their village was presented as the only way to increase use of facility care. Some expressed frustration that antenatal consultations were only available in the morning, thus making it difficult to attend. Using the childbirth services demanded effort from women and their families; having to walk for several hours and being away from home for more than 12 hours were commonly reported. Sometimes these efforts did not result in care at the health centre:

‘Since we live far from the health centre, when we arrive for the consultation [ANC], they tell us that we arrive too late. They don’t want to work. They say that the sun is high and when the sun is high the bleedings don’t stop. They don’t do the weighing [ANC], and you return home. Another day, you go to the health centre again, and they refuse you once more. They will not wait for you. They bother us with that. We get tired.’

Woman, FGD 3

Common concerns regarding the care provided at the health centres were the waiting time for the antenatal consultation, and the availability of water, lighting and medications. A minority of the participants were clearly dissatisfied with the pregnancy and birth care provided at the health centre. One concern was confidentiality: giving birth at the health centre would lead to the community knowing how long the delivery lasted -having a quick delivery was seen as a sign of pride in the area. Others raised the issue of disrespectful treatment by health staff and that some health workers would say that the women smelt badly, made fun of their suffering or neglected the women by simply leaving them to themselves during birth.

‘I gave birth at the health centre. They did not care for me… When I arrived at the health centre I found the health worker. He said it was the time of birth. He gave me a bed, then he closed the door and left. I did not know where he was. I gave birth alone.’

Woman, FGD 1

To avoid such disrespectful care at the health centres, different measures were taken by communities. In one village, family members were sent out to see which health workers were on call before going to the health centre for care. Another example was how women chose to give birth at home, then brought the baby to the health centre immediately afterwards saying that the delivery was quick. While the two previous examples illustrate strategies and agency to influence the care received, one father explained how conflicts with health workers over costs after home births led him to not bring his wives at all to the local health centre. When asked about other possible ways to influence the care received at the health centres, some participants stated that they feared complaints directed at health workers would have negative consequences for their future treatment at the health centre.

‘If I say that you’re not doing a good job, and if I’m the only one? to say so, the day I come for help, will you accept to care for me?’

Woman, IDI 10

## Discussion

The study findings illustrate how facility based pregnancy and birth care was the dominant discourse, how the use of childbirth services was perceived to be enforced through the use of sanctions, and how community members, to a large extent, remained incapable of questioning facility-based practices. We will now proceed to the discussion, starting with examining MDG 5 on maternal mortality reduction as a global social action. Second, we will explore how the experience of sanctions at the health facilities has negative consequences for trust in the health system. Third, we will argue that the pressure to use facility pregnancy and birth care contributes to the further marginalization of women with poor access to facility care.

### Promotion of institutional care as a global social action

Global policies articulate with national and local policies and practices in Burkina Faso, where the MoH has as an expressed ambition to ‘Contribute to the achievement of the MDGs by accelerating the reduction of maternal and neonatal mortality’ [14]. The country has implemented the strategy to have a skilled provider attend 80% of all births, with the aim of reducing maternal mortality. In the study area, attending at least four antenatal care consultations and having a facility birth were heavily promoted by health workers. These services were described as essential by users, and are both used as indicators for the progress towards MDG 5 [3]. These will probably also measure progress towards the newly adopted Sustainable Development Goals (SDGs), although the list of indicators has not yet been approved.

Merton’s concept ‘imperious immediacy of interest’ can help us unpack the effects of the promotion of facility pregnancy and birth care reported in this study [10]. The urgency of addressing preventable maternal deaths on global, national and local levels constitutes an immediacy of interest. Policymakers’ and health workers’ preoccupation with increasing the number of institutional births to reduce maternal mortality may divert attention away from other consequences of the policy and its implementation. The aim of increasing the number of women who give birth in a health facility and the methods employed to achieve this goal seem rational when seen in isolation. However, actions to increase institutional births may diverge from other important principles in public health, and with local values in the communities in which they are implemented, such as health system trust and equity. As the discussion proceeds, we will explore the unintended consequences of the promotion of institutional pregnancy and birth care by examining the values and interests affected in a rural Burkinabè context.

### Unintended consequences for health system trust

Sanctions against women not using the health services as expected by health workers constitute a distortion of the global and national institutional birthing policy. It has already been reported how the demand for improving performance indicators in the field of maternal health makes health workers use persuasive and coercive strategies to increase facility care [12,28,29]. We propose that health workers’ reported eagerness to increase the utilization of pregnancy and childbirth services is an expression of the pressure put on the entire Burkinabè health system to ‘Contribute to the achievement of the MDGs’ [14], and that the fear of sanctions plays an important role when women decide to seek facility care. The use of sanctions raises normative questions about the role of and inherent values in health systems. Use of facility pregnancy and birth care seems to have become an imperative rather than an offer, and health workers are seen by communities as enforcers of an unwritten law of institutional care. Users feel that they are left with no choice but to comply with health workers’ recommendations to receive proper care and avoid sanctions.

Home births remained prevalent in the study area although institutional care emerged as the only acceptable option. According to government data Burkina Faso had an estimated home birth rate of approximately 25% in 2010, which might mean that home births are not solely the result of constraints, but also of preferences for traditional practices and/or dissatisfaction with treatment in health facilities [17, 23, 30]. A recent study from Zambia illustrates how difficult it is for communities to challenge the official norm of institutional care by saying that they prefer home births [31]. Women’s reported satisfaction with, and preference for, facility pregnancy and birth care during interviews could be understood as an expression of the dominant discourse on institutional births, political correctness or passivity [32,33]. However, their expressed preference for facility care may also be associated with a belief in health facility as the safest place to give birth in medical terms and thus preferable, despite concerns about staff attitudes.

The asymmetric distribution of power between the poor illiterate women and the more educated healthcare providers may create norms of passivity, where users of birthing services do not declare their dissatisfaction, even in cases of negative experiences [34]. In the few cases where community members contested sanctions or expressed discontent with the services, they feared conflicts with local health workers. Community members are largely dependent on the services provided by the local health centre, and they stand to lose their only access to health care if they challenge the local power structure [35]. Our findings suggest that the sanctions experienced by women and their families may work to increase institutional delivery among women with easy access to health services, but may also undermine health systems trust and further worsen access among already marginalised pregnant women.

### Social suffering as an unintended policy implication

Actions that could be aimed to increase the use of services might become, in this context, obstacles to service utilisation for women with already poor access to care. Although the sanctions may have been locally implemented to increase the use of pregnancy and childbirth services, their complexity may nevertheless have the opposite effect. Aïcha reported not giving birth at the health centre due to the fear of verbal sanctions. Her baby died within hours after a home delivery lasting for more than a day. Although the cause of her baby’s death, and her reasons for not seeking care remain complex, Aïcha’s story raises questions of whether the practices aimed to increase the use of facility services and reduce maternal and newborn mortality exclude, rather than include, women in need. Maternal deaths are largely influenced by social context and young, rural, illiterate single mothers are at significantly higher risk of poor pregnancy outcomes [36,37]. Excluding already marginalized women constitutes an unforeseen and unwanted effect of the institutional birthing policy, where actions to increase the numbers of women receiving care in facilities conflicts with the principle of promoting health equity. This resonates with Merton’s concern that the ‘imperious immediacy of interest’ may in unintended ways defeat the attainment of other fundamental values within communities and institutions [10]. We will, through Aïcha’s story, now discuss how the pressure to institutionalize births may contribute to social suffering.

The social suffering framework helps us explore how political, economic, and institutional power influences access to care, and how these types of power impact policies and actions implemented to increase institutional births in the study area [11]. The framework takes particular regard to the poor and marginalized groups, which are, by historical and social disparities placed at the back of the line for high quality care [38]. We argue that sanctions may worsen health outcomes for the already marginalized in the name of improved maternal health. Due to existing power structures between mothers and providers, communities are unable to influence the use of sanctions; their only way to prevent bad treatment and/or economic sanctions is to withdraw from the health system. This is exemplified by Aïcha, as well as by the other women who choose to arrive just after delivery or not attend the childbirth services if certain health workers were on call. The fear of economic sanctions may even prevent communities from seeking care in case of emergencies. The use of sanctions will, in this way, lead to poorer access to health care for the women and infants in question, and worsen rather than improve their health outcomes. The pressure to institutionalize care may thus exacerbate health problems for women with limited access to facility care.

The sanctions described in our study may lead to worsened social outcomes for communities that already have poor access to services. For many rural women, like the participants not reaching the ANC clinic by foot before closing time, the non-use of facility services does not represent an intended choice. Rural women often cannot influence the factors constraining them from using the services as prescribed by health workers, namely distance to the health centre, financial constraints and limited decision making power within the household [39]. The perceived use of sanctions by health workers was not accompanied by any efforts to overcome access barriers, and could reflect an overestimation of women’s agency. Furthermore, it implies blaming women for their poor health outcomes. Such practices of placing the ‘responsibility’ for ill health on the individual influences community perceptions about the ‘right place’ to receive care [40]. Economic sanctions, as practiced in the study settings, could further impoverish families not accessing care for various reasons. Imposing sanctions on women for factors they are unable to influence adds an unintended double burden with social stigma and/or increased financial constraints to the burden of not accessing health care.

For the study participants, frontline health workers are represented as the implementers of an institutional care policy with serious unwanted effects such as the sanctioning of women with poor access to care. However, it is important to keep in mind that the health workers in question operate within a health system of severe constraints under the pressure of a strong international discourse on institutional births. As Kleinman pointed out, the potential for harm lies latent in institutional structures, such as health systems, that are authorized to implement social actions. Even though the separate health centres remain the sites where unwanted effects are operationalized, it is the responsibility of policymakers both on a global and a national level to propose actions to reduce the unintentional harmful impacts of policy [11].

### Methodological concerns

This study reports from four health centres in a rural part of Burkina Faso. Although the centres were purposively selected based on their diversity in terms of location, size and assisted birth coverage, one cannot rule out that there might be systematic differences between the study health centres and other health centres in the two districts. However, we do believe that the findings are also relevant in other primary health care settings in Burkina Faso.

AM, collected the data for this paper as part of her doctoral study, in each of the four primary maternity units. She did not provide patient care, but her and the research assistant’s participation in clinic activities at the health centres might have contributed to her being perceived as a health centre representative, which may have biased participants’ responses. The interpretation of the findings may have been influenced by AM’s position as a European medical student, coming from a very different health system and culture in which patient rights, user involvement and choice are basic values. While throughout the research process AM tried to ‘bracket’ these values and remain open to the local conditions for health worker-patient interaction, her background and values may have made her particularly attentive to the adverse effects of the strong discourse on institutional delivery, and to the pressure to which individual women were exposed.

## Conclusion

This paper documents that women in rural Burkina Faso and their families experience a substantial pressure from health workers to secure health facility childbirth. We suggest that the use of health facility at birth has been transformed from a recommendation to an obligation since women are negatively sanctioned if they do not comply. We argue that this is a perversion of the goal to provide skilled attendance at birth, and that it moves responsibility to use health facility at birth from the health system to the individual woman with limited power to overcome access barriers. Our study indicates that the implementation of the policy of skilled attendance at birth in Burkina Faso may undermine the trust in the health system and may counteract the overall goal to reduce maternal mortality for certain groups. However, the current study’s exploratory approach does not allow us to draw conclusions on the prevalence of these negative impacts. The findings call for an increased research focus on, and policies to address, the unintended local consequences of the preoccupation with institutional births as we enter the SDG era.

## Supporting Information

S1 Interview Guide Interview guide: Women with a recent facility birth. (DOCX)

S2 Interview Guide Interview guide: Women with a recent home birth. (DOCX)

S3 Interview Guide Interview guide: Focus Group Discussion. (DOCX)
