# Supplementary material for: Reflections on the Unintended Consequences of the Promotion of Institutional Pregnancy and Birth Care in Burkina Faso
Source: PLoS One. 2016 Jun 3;11(6):e0156503. doi: 10.1371/journal.pone.0156503 (PMC4892534; doi:10.1371/journal.pone.0156503)
Supplement: S1 Interview Guide — (DOCX) [file pone.0156503.s001.docx]

Interview guide: Women with a recent facility birth

Basic demographic data:

Basic demographic data should be collected during an informal conversation to establish privacy reports.

Name of the woman: .........................................................

Village of residence: ....................................................

Age (years): / _____ / Gravidity: / ____ / Parity / _____ /

Number of living children: / ____ /

Number of children born alive and deceased: / _____ /

Number of spontaneous abortions or stillbirths: / ____ /

Age oldest living child (years or months): / _____ /

Age of the youngest living child (years and months): / ______ /

Marital status: /_____/

Your husband, has he other wives? yes / ____ / no / ____ / If yes how many? / ____ /

What is your profession?

Do you have a gainful activity? Yes No /__/

What is your highest level of education? / _____ /

Have you been literate in any language?

If so how long (months): / ____ / and in what language? ......................

Date of your last birth: / ____ / _____ / _____ /

This discussion is part of a study to better understand how we can improve the health of mothers and babies in this region. We ask you to share your thoughts on the practice at the time of delivery.

In this region there are women who deliver at home and there are women who deliver at the CSPS or hospital. During your pregnancy, could you talk about the different choices on the place of birth with your family members. If that was the case, can you tell me the course of these conversations?

• Which family member initiated these conversations?

• Do you remember the different options about the place of your birth?

• Do you were all in agreement, or were there disagreements during these conversations?

• In case of disagreement on the place designated for delivery, which member of the family took the final decision on the birth place?

• Why did this family member make the decision?

• How did you feel about this decision?

• If it was up to you, which place would you choose? Why?

• Did you give birth in the decided place, or was there a change of venue?

• Why were there a change of venue?

• Who assisted you during birth?

Many women go for antenatal care during pregnancy to check that all is well with mother and baby. Have you made at least one weighing (ANC) during this pregnancy?

Can you tell me your experiences during these consultations during this pregnancy?

• If yes, how many times did you go to the CSPS? In which months of pregnancy?

• How did you experience these consultations? What kind of care did you receive? How would you describe the care received?

• If you did not attend ANC in the CSPS, why?

• Did you go outside the CSPS for care during your last pregnancy? Where? Why?

Can you tell me where, how and when the delivery began?

• When did you feel the first uterine contractions?

• Who was with you at that time?

• Members of your family, were they with you?

• Can you tell me who were there?

How did you make the decision to go to the health centre?

• At what stage of labour did you go?

• At what time (morning, afternoon, evening, night)?

How did you come to the CSPS?

• Which type of transportation?

• How much did you pay for transport?

• Did anyone accompany you? Who?

• Did you have problems finding transportation?

• How much time went by between the decision to go to the health centre and your arrival there?

Arriving at CSPS, how were you received by the staff?

• How long did you wait before getting care?

• Who took care of you while waiting?

• Is this waiting time acceptable for you?

What kind of care did you receive during birth?

• What was done to you and your baby?

• How did you appreciate this care?

• Your expectations for care, have they been addressed?

• According to you, what was good about the care received?

• What was missing from the care received in your opinion?

• Where did you give birth?

• Were you afraid that someone else could hear you during birth?

Did you receive enough information during childbirth?

• Did health workers explain why they examined you?

• Did you feel calm when health workers examined you?

• Did you have the opportunity to ask questions to health workers?

How did health workers ensure your comfort during childbirth?

• Could you call health workers during childbirth?

• Who was with you at the time of birth?

• Who was with you during the birth of the placenta (third stage of labour)?

How did health workers treat the family members present at birth?

• Did they provide them with enough information on the progress of birth?

• Did they ask for money before birth?

• Were members of your family allowed in the delivery room?

In case of evacuation to Banfora hospital:

Can you describe evacuation to hospital Banfora?

• How long did you stay at CSPS before evacuation to the hospital?

• How was the referral decision taken?

• What means of transportation was used?

• Did you have problems finding transport?

• How was payment for transportation made?

• Did anyone accompany you?

• How long did it take before your arrival to Banfora?

• This time, is it acceptable?

Childbirth can be subject to a payment.

How much did you pay for the care provided during this last pregnancy?

• For antenatal care?

• For medications?

•For transport?

Did you pay for the care provided during childbirth?

If so, where and to whom?

• What is the cost of delivery to the CSPS?

• What is the cost of a birth attended by a village midwife?

• How do you know the cost?

• The price, was it decisive for the choice you made?

In total, how did you or your family pay for this delivery?

• For medications?

• For other things?

• Did you have difficulties to pay this/these amounts?

• How did you pay for this amount (savings, sales, loans)?

If other children:

Can you compare your last birth to your other deliveries?

What is your general impression of the care you received during birth?

• Do you recommend facility birth to the other women in your community?

In general, what do the people in your locality think of health centre childbirth?

• What do women think?

• What do men think?

And yourself, what do you think?

In general, what do the people in your area of ​​home birth?

• What do women think?

• What do men think?

And yourself, what do you think?

What type of intervention can be put in place to improve your preferred place of birth (CSPS or home)?

What type of intervention can be put in place to reduce deliveries at the location (home or CSPS) that you do not prefer?

Do you have anything else you want to add?

Thank you very much for your contribution!
