# Supplementary material for: Reflections on the Unintended Consequences of the Promotion of Institutional Pregnancy and Birth Care in Burkina Faso
Source: PLoS One. 2016 Jun 3;11(6):e0156503. doi: 10.1371/journal.pone.0156503 (PMC4892534; doi:10.1371/journal.pone.0156503)
Supplement: S3 Interview Guide — (DOCX) [file pone.0156503.s003.docx]

## **Interview guide: Focus Group Discussion**

*This discussion is part of a study to better understand how we can improve the health of mothers and babies in this region. We ask you to share your thoughts on the practice at the time of delivery. Please do not tell other members of the community that will be said in this discussion. Beginning I please remember your name, age, place of residence and the number of children you have.*

| Name | Gender | Age | Number of Children | Village of residence |
| --- | --- | --- | --- | --- |
|  |  |  |  |  |
|  |  |  |  |  |
|  |  |  |  |  |
|  |  |  |  |  |
|  |  |  |  |  |
|  |  |  |  |  |
|  |  |  |  |  |
|  |  |  |  |  |
|  |  |  |  |  |
|  |  |  |  |  |

I would like to start the discussion by asking questions about the preparations for delivery by the pregnant woman and her family.

When a woman is pregnant, is there a discussion on the place of birth in:

-the couple?

-the family?

• What are the different places one could give birth?

• Which family member initiates these conversations?

In this region are preparations for childbirth being made during pregnancy (transport, savings)?

• What kind of preparations are made for the woman?

• What kind of preparations are made for baby?

• What preparations are made by the wife / husband / the tanties / stepmother?

•During which months of pregnancy are these preparations made?

In case of disagreement on the place designated for delivery, which member of the family makes the final decision on the place of birth?

• Why does this person make the final decision?

Women give in different places -In the village or at the health centre. I would now like to ask you questions to better understand why women deliver in different places.

What are the reasons women give birth at home?

• What measures are taken by the other family members to help the woman in labour?

• What are the benefits of a home birth for the woman and her family?

• What are the inconveniences of a home birth for the woman and her family?

How do village midwives assist women in childbirth?

• Do village midwives assist women come home?

• What are the benefits of a birth attended by a village midwife for the woman and her family?

• What are the inconveniences of a birth attended by a village midwife for the woman and her family?

Does health workers assist women in childbirth in their homes?

• How does one receive their assistance at home?

What does health workers to assist women in childbirth at health centres?

• How are people who accompany the woman treated?

• Do they ask the woman or her family to procure consumables (or birth kit)?

• What is the price of a simple delivery / delivery with episiotomy at the health centre?

• What happens when a woman can not afford to pay for delivery?

• What are the benefits of a health centre birth for the woman and her family?

• What are the inconveniences of a health centre birth for the woman and her family?

In this region, husbands might be absent at the time of delivery.

• Where would they be?

• What happens if the husband is absent at the time of birth?

• Can a woman go to the CSPS without the consent of her husband?

Some women have complications during pregnancy, childbirth or during the weeks following childbirth. I would like to better understand what happens in these cases.

In this region, there is what kind of health problems can a woman encounter?

• During pregnancy?

• During childbirth?

• During the weeks following childbirth?

In your opinion, among these problems, what is the most serious?

• Do you know of women who have had any of these problems?

• What were the reasons for this problem?

• What happens to women who have these problems?

If a woman in your village is subject to a problem during delivery, what can she / her family do?

• What steps can be taken at home / in the village?

• Where can go to receive the necessary care?

• How are women with problems cared for within the health centre?

Do pregnant women and / or their families prepare for the occurrence of a difficult birth?

• What could be the content of such a preparatory plan?

• Which family members do / should do these preparations?

We talked about the preparations for the birth, and the progress and normal deliveries and complicated deliveries. Now I want to ask you about the period following childbirth.

Does someone visit the mother after delivery?

• Who?

• How many days after birth?

• Is there any particular day to visit?

• Are there days or the visits are prohibited?

• Are there people who are not allowed in the house of a woman who has just given birth?

After childbirth, is the woman is free to leave her house as she wants?

• Why / why not?

• If not, after how long can she leave her house?

According to you, should women go to the health centre after delivery to ensure that all is well with her and the newborn?

• How long time after childbirth?

• If everything seems to be going well, is it necessary to go to the health centre?

• Who can accompany the mother and baby to the health centre?

Finally, I want to ask you questions about your perception of the health centre in your village and the care received there.

Some health centres have days dedicated to the ANC, days for immunization days for family planning. Is that appropriate?

• If not, what (s) day (s) of the week you prefer? Why these days?

In general, what do the people in your locality think of health centre childbirth?

• What do women think?

• What do men think?

Is that in the last five years, have there been a change in the prices for different health centre services:

• ANC?

•ANC booklet ?

• Childbirth?

• Consultations?

In case of new prices:

These new prices, have they influenced attendance at the:

• ANC?

• Childbirth?

• Consultations?

In general, what do the people in your area think of ​​home births?

• What do women think?

• What do men think?

What type intervention can we implement to improve ANC?

What type intervention can we put in place to improve birth care at the health centre?

What type of interventions can be implemented to improve consultations?

What type of intervention can be put in place to reduce home births?

Thank you very much for your information. We believe that the experiences and thoughts you've shared with us will be very useful to improve maternal health and child health Banfora.
